# The Five Books of Theophilus Protospatharius on the Construction of the Human Body

**Published:** 1843-04

**Authors:** 


### Art. XII.

*Θεοφιλου Πρωτοσπαθαριου περὶ της του ανθρώπου κατασκευης, βίβλια ε.*

*Theophili Protospatharii de corporis humani fabrica, Libri V.* Edidit Guilielmus Alexander Greenhill, m.d.—*Oxonii e Typographæo Academico,* mdccclxii.

*The Five Books of Theophilus Protospatharius on the Construction of the Human Body.* Edited by William Greenhill, m.d.—*Oxford,* 1842. 8vo, pp. 444.

It so seldom happens that we have to announce to our readers a new edition of an ancient medical work from the press of this country, that we are induced to devote more consideration to the publication at the head of this article than we would otherwise have been disposed to bestow upon it. Indeed, since Wigan's edition of Aretæus, Oxon. 1723, as far as we can recollect, no edition of any Greek medical author, and certainly none that has acquired any considerable share of literary celebrity, has been published in Great Britain. This can be accounted for only by the indifference with which the ancient authorities in medicine have been regarded of late by the profession in general; and this neglect has now become so great as sometimes to manifest itself in a way which we cannot but apprehend must exhibit the present state of our medical literature to our continental neighbours in a very unfavorable light. To give an example which presently occurs to us: Sir Astley Cooper states in his Surgical Lectures that in former times, (meaning seemingly in the days of his immediate predecessors,) the medical profession was utterly unacquainted with dislocations at the hip-joint; and he makes mention of a surgeon in London about fifty years ago, who denied the possibility of such an occurrence. The inference which seemingly he wishes to be drawn from this statement is, that all the important information which the profession is possessed of relative to this class of accidents has been derived entirely from himself and his contemporaries; and if this was his object, it must be admitted that he has been very successful in attaining it; for we have seen it repeatedly stated in the journals of the day, among the important improvements in surgical practice introduced by this surgeon, that he has the merit of having first made the surgeons of London acquainted with dislocations at the hip-joint. But the assertion of Sir Astley Cooper must surely have been rashly made, and upon some misapprehension. For we cannot bring ourselves to believe that the magnates of our profession in London can ever have been so destitute of all proper acquaintance with preceding authorities, or have been so miserably deficient in all talent for practical observation, as we must suppose them to have been if the statement made by Sir Astley had been well-founded. In fact we have every reason to think that at no previous period did the neglect of ancient authority prevail to so great an extent as at present, and that at no other time would this assertion of Sir Astley's been passed so long without exposure. Be that, however, as it may, it ought to be generally known, that the four common varieties of dislocation at the hip-joint, namely, *outwards* on the dorsum of the ilium, *inwards,* upon the thyroid ligament, *backwards* upon the tuber ischii, and

*forwards* upon the ramus of the pubes, are as well described by Hippocrates, and after him by Galen, Oribasius, and Paulus Ægineta among the Greeks; by Celsus among the Romans; and by Albucasis, Rhases, Haly Abbas, and Avicenna among the Arabians, as they are by Sir Astley Cooper himself, or by any other medical writer of the present day. If the reader will consult the Venetian edition of Galen's works, or the edition of Oribasius, published among the *Medicæ artes principes* by H. Stephens, he will find drawings of these dislocations executed with no contemptible degree of accuracy. Thus much in regard to the importance of cultivating an acquaintance with the ancient literature of our profession.*

The Greek treatise of Theophilus was first discovered about the year 1536 by Franciscus Frigimelius, and was published in Latin at Venice the same year by his pupil, Paulus Crassus, a scholar of some eminence in those days, and distinguished as the translator of several other medical authors. This edition was reprinted several times and at different places during the following thirty years. The celebrated printer, Morel, first published the original at Paris in 1555. This edition is executed in very elegant characters; but as it contains neither Latin interpretation nor any other subsidiary information, we need scarcely say that it is not well adapted to the taste of the present age. The Greek text of Morel, and the Latin version of Crassus, were inserted, along with a preface, by Albertus Fabricius in his Bibliotheca Græca. These were the only printed editions of the Greek original previous to the Oxford edition now on our table. Four MSS. of the Greek text exist; three in the King's Library at Paris, and one in Venice. Of the Parisian MSS. the first is the only one of any great value, and is the one from which Morel is supposed to have printed his edition. The Venetian MS. is of a decidedly superior character, and has therefore been chosen, with much judgment, for the basis of the present edition by Dr. Greenhill; but he has taken great pains further to improve the text, by collating it with the other MSS. and previous editions, and also by comparing the text with that of Galen *De usu Partium*, from which a great portion of the work in question is taken; and further, where all manuscript authority failed to ensure a sound text, Dr. Greenhill has in various instances attempted to improve it by his own conjectural emendations and those of two literary friends, whose assistance he gratefully acknowledges, (Ed. Mon. p. vi.) Moreover, he has given the prefaces of Crassus and Fabricius to their editions, and that of Andreas Mustoxydes and Demetrius Schinas, two modern Greeks, who published fragments of the author at Venice in 1817. The text is throughout illustrated by marginal references descriptive of the matters treated of; and in these the editor, by adopting the modern terminology, has thereby contributed an essential service to the student, who can thus have no difficulty in discovering the anatomical parts to which the descriptions are meant to apply. But by far the most laborious and difficult part of the editor's task must have been the compilation of the *Adnotationes*, which contain a rich mine of critical and scientific information, and therefore cannot fail of being eagerly explored by those

* We hope the Sydenham Society just founded in London under the most favorable auspices, the main object of which is the republication of the ancient medical classics and the select works of the older moderns, will tend to do away this opprobrium of our age.

who are minutely acquainted with and take an interest in the ancient literature of our profession. To a sufficiency of verbal criticism are added brief observations on the anatomical and physiological subjects treated of by the author. But however much we may admire the industry and ability displayed by Dr. Greenhill in this part, we must decline attempting to give our readers an analysis of its contents, which do not well admit of being presented in a condensed form, and are not well adapted to quotation in such a work as this. Our general opinion of the edition we may give in a few words: that taking into account the pains bestowed upon the accuracy of the text, with the elegance of the typography, and the value of the annotations, we look upon this as being decidedly the best edition of an ancient medical author with which we are acquainted.

We shall now proceed to give our readers some account of THEOPHILUS and the matters treated of in his work. Regarding the author himself little or nothing can be ascertained. It is not even agreed to which century he ought to be referred; only this far is discovered from the language of his work, which contains several barbarous words not used by the writers of the earlier centuries of the Christian era, that he cannot be supposed to have flourished before the seventh century. His quotations from the Septuagint and New Testament, together with the peculiar style in which he mentions a Supreme Being, (B. i. § ii.) leave no doubt that he must also have been a zealous Christian. There are also such traces of medical knowledge in this work, and still more in his Commentaries on Hippocrates, published a few years ago by Dietz, (Koningsburg, 1834,) that we need have little hesitation in setting him down for a practising physician.

The work now under consideration, entitled "On the Construction of the Human Body," in Five Books, is little else than an abridgment of Galen's celebrated treatise, "On the Uses of the Parts," in Seventeen Books. The object of it is to show forth the wisdom of God in the formation of the human body. In a word, what a considerable portion of Dr. Paley's work on Natural Theology is to the religious reader of the present day, such must Galen's great work, or the elegant abridgment by Theophilus, have been to those of former times. And indeed the original work of Galen need not fear a comparison with the modern; whether we regard the elegance of its style, the acquaintance with minute anatomy which it displays, or the profound system of rational theology which it inculcates.

Throughout this treatise Galen, in imitation of the great peripatetic philosopher in his work "On the Members of Animals," has advocated the doctrine of *final causes*, contending that *ends* procure *means*, instead of holding with the Epicureans and their modern copyists of the French school, that means led to ends. "It is not," says Galen, "because man has hands that he is the noblest of animals, but because he is the noblest of animals therefore he has got hands. For it was not hands which taught man the arts but reason; hands being the instrument, as the harp is of the musician, and the tongs of the smith (*De U. P.* i. 3.)"* And in the following passage, which we could have wished to have found in Theo-

* Sir Charles Bell coincides with Galen in this view of the subject. (Bridgewater Treatise, Chap. x.)

philus, Galen thus unfolds his views with regard to an immaterial principle in animals:

"The use of all *the members* is for the sake of the soul; for the body is its instrument, and on this account the members of animals differ much from one another, since it is so with their souls. For some of them are courageous and some timid; some are savage and some tame, some are, as it were, political and mechanical; and some, as it were, solitary. The body then is suited to all the affections and powers of the soul; to the horse it is equipped with strong hoofs and a mane, for he is a swift, a proud and not a spiritless animal. To the lion, as he is passionate and strong, it is strengthened with tusks and claws; and thus to the bull and the boar, to the one horns and to the other prominent tusks are the natural arms; but to the stag and the hare as being timid animals, the body is entirely naked and unarmed. For, no doubt, swiftness suited the timid and arms the strong. Nature then has furnished no timid creature with arms nor has left any bold one naked. But to man, as being a wise animal, and the only divine one upon the earth, instead of all other defensive arms nature has furnished HANDS, an instrument necessary for all the arts, both of peace and of war." (*De U. P.* i. 12.)

From this extract it will be seen that in ancient physiology mind or soul was a term of wide signification, and that to its operations were assigned many offices which would be looked upon as purely material according to the prevailing ideas of the present times. Indeed the ancient physiologists had more comprehensive conceptions of mind than we have, (whether or not they were more accurate we shall not venture to affirm,) and recognized traces of its activity in various phenomena which we usually ascribe to *laws* originally impressed upon matter, and operating in some manner which we do not pretend to explain. Thus, besides the DIVINE MIND, which they held to be pure reason unmixed with matter, the Platonic philosophers recognized mind as the active principle in the human organism, and not only in man but also in all the inferior animals, from "the half-reasoning elephant," down to corals, sponges, and other zoophytes; nay, in vegetables and all organic bodies they held that there is an innate principle of motion, an active and immaterial essence, the cause of all internal changes, to which they gave the name of NATURE.* According then to this philosophy, nature is mind, diffused through all the works of creation, performing certain operations, and acting according to certain determined laws, but without consciousness. Though ever operating with consummate skill, the wisdom of its operation is not of itself, but derived from the FIRST CAUSE which created it and infused it into the universe. It is to be remarked in this place that Galen usually ascribes to nature those functional operations in the human frame which his Christian epitomizer attributes to the immediate agency of the Divinity, and which a material physiologist would refer to the laws of vitality or of organization. It is not difficult then to see that by the vegetative life of plants and the nutritive life of animals the ancient physiologists meant the energy of a portion of that principle which we are treating of. "The nutritive part of the soul," says Aristotle, "performs its operations in sleeping, more than in waking; for then, more than at other times, are animals nourished and grow in bulk, as they have no need of sensation for these purposes." (*De Somno,* c. i.)

* The original source of the philosophical doctrines here expounded may be found in Aristotle's *Auscultationes Naturales.* See in particular Simplicius's Commentary on the second book. A brief exposition of it may be found in Nemesius *de Naturâ Hominis.*

Altogether, as we have stated above, the ancient physiologists held that the only active principle in our body is *mind*, and that the corporeal organs and members are the instruments by which it performs its operations. According to this philosophy it was *for* mind that the great ARTIFICER* constructed the fabric of our body, and *by* mind that the body preserves its organization during life. For, as Hippocrates felicitously expressed it in figurative language, the elements of the microcosm, or little world of the human frame, are engaged in a constant warfare with those of the macrocosm, or the great world, and it is mind which enables the body to maintain this struggle. From what has been already said we will be able to comprehend what the ancient philosophers meant when they likened *mind* to *form*, the body being looked upon as owing its configuration entirely to mind. These doctrines, originating in the Platonic and Peripatetic schools of philosophy, were zealously advocated by Galen, and traces of them, modified no doubt by the principles of his religious faith, are to be found in Theophilus.

We stated so fully in our last Number our own opinions respecting vital properties that we need not occupy the time of our readers in pointing out wherein we differ from Galen. Enough has now been said by us to show that Galen and the physiologists of antiquity knew fully as much about the matter as any of our modern advocates of a vital principle. And, by the way, Lord Brougham, who in his recent edition of Paley's Natural Theology, charges his author with overlooking the importance of mind in the microcosm, would have found Galen, in his work "On the Uses of the Parts," fully as *spiritual* as even he could desire.† And with regard to that principle in the universe which the ancient philosophers call nature, we refer such of our readers as wish to prosecute the subject without being subjected to the trouble of studying Aristotle and his Alexandrian Commentaries, to Cudworth's Intellectual System, where they will find the ancient doctrines fully expounded and illustrated. The ancient principle would seem to be identical with what the German metaphysicians call "the unconscious reason of the universe."

Connected with this subject is the ancient classification of the faculties of organized bodies, alluded to very obscurely and indistinctly by Theophilus in a passage (l. 3,) where the text may justly be suspected of being corrupt. The faculties, then, were divided into the natural, the animal, and the vital. Under the first were comprehended all those faculties or energies common to animals and vegetables, which are subservient to generation and nutrition. To the animal were referred those of loco-

* This term δημιουργὸς is regularly applied by Theophilus to the Divinity. It is not, however, of Christian origin, as we have seen stated, for it occurs also in Galen's work, and in fact it can be traced to the *Timæus* of Plato.

† The learned Dr. Turton, in his work on Natural Theology, comes to the rescue of Paley, and maintains that there is no foundation for the charge which Lord Brougham brings against him. We must say, however, that it strikes us very forcibly that, according to Paley's system, mind does by no means play so important a part in the animal economy as that which is assigned to it by Galen and Lord Brougham, for his lordship, without seeming to be aware of it, is but the reviver of the Galenical doctrine respecting the functions performed by an immaterial principle in the bodies of animals. Lord Brougham is a follower of Plato, and Paley of Anaxagoras, concerning whom Socrates complains in the *Phædo*, that he sets out with assigning mind as the cause of all the phenomena of the universe, but when he comes to particulars he accounts for everything without the agency of mind.

motion and sensation; and to the vital were referred those by which the higher classes of animals preserve an equability of temperature, namely, those connected with respiration. The reader who chooses to prosecute the subject further will find it treated of at great length by Galen in several distinct treatises, and more methodically by Haly Abbas, the Arabian. (*Theor.* lib. ii.)

We must also in this place allude to the distinction of the causes of things first introduced into philosophy by Aristotle and noticed prominently by Theophilus in one passage. (Lib. iii. 12.) We shall give it as it is laid down by Aristotle's paraphrasist, Andronicus Rhodius; "There are four causes of things, the final, the formal, the material, and the efficient. The efficient is the energy of the artificer; the material is the wood and stone of which the house consists; the formal is the form or plan of the house; and the final is that for which the house was made." (*In Eth. Nic.* i. 85.) Theophilus thus applies this arrangement to the case of the human voice:

"Since man speaks and talks, and the accomplishment of everything that takes place in man is from causes, and the causes are four-fold, efficient, formal, material, and final: the efficient cause of the human voice may be held to be the brain, moving the chest voluntarily, (the proximate cause of the voice indeed is the brain, for the remote is the soul directing the brain to move the chest: and then, since the efficient cause is taken in a double sense, the proximate and the remote, there may be reckoned in the case of the voice a still more remote cause, that is to say, the cause of all things, and comprehensive wisdom of God which thus formed and fabricated the members of man;) the material is the air which rushes forth in expiration; the formal, is the wind-pipe and the coat common to it and fauces; and the final, the voice itself."

The late Professor Dugald Stewart, speaking of the Aristotelian arrangement of causes, joins his countryman Dr. Reid in holding "that it amounts only to an exposition of the different meanings of an ambiguous word." Now, without stopping to examine whether or not there be any room for the censure here implied, we shall only remark that we have seldom occasion nowadays to complain of authors who treat of abstract subjects in physiology that they take too much pains to define the terms which they make use of. Our editorial labours would be much lightened if our present dabblers in abstract questions regarding vitality would follow the example of Aristotle and Galen in setting out with a well-defined terminology.

We shall now proceed more in order, to give our readers a brief abstract of the matters treated of in the five books of Theophilus.

The remainder of the First Book after the first two chapters on preliminary matters, is devoted entirely to the describing of the mechanism and uses of the different parts which compose the *hands* and the *feet.* It forms a very lucid and useful compendium of the first three books of Galen's work "On the Uses of the Parts." This portion of his work has been so often translated and commented upon in modern treatises on Natural Theology, that we cannot but suppose that most of our readers must be more or less acquainted with it; we do not think it necessary then to give any analysis of it, and indeed it would be no easy matter to do this, seeing it is almost entirely made up of minute descriptions and reflections on mechanical contrivances, so as not readily to admit of abridgment. We might have felt a disposition to compare this treatise of Galen's with the Bridgewater Treatise of the late Sir Charles Bell, "On the Hand." But here we should find ourselves at a loss to institute any particular comparison between two works bearing scarcely any similitude to one another. Galen has all along stuck close to his subject, and has done such ample justice to it as to leave little prospect to a subsequent labourer in the same field, of being able to produce anything strikingly original, provided he pursued the same path of inquiry. Indeed it has often struck us that Sir C. Bell, must have felt strongly this inconvenience attending his task, and that this is the reason why he indulges in digressions into all sorts of collateral subjects, some of which bear but very remotely upon the one which he had undertaken to illustrate. Altogether then we look upon the English work, although rich in facts deduced from geology and comparative anatomy, two sciences which can scarcely be said to have had an existence in the days of Galen, as far inferior in interest to the other, if viewed as a disquisition on the adaptation of mechanical contrivance in the hand, to the purposes for which it was intended. And with regard to ability for grappling with abstract questions in philosophy involved by the subject, there can be no doubt but that Galen was just as much superior to Sir Charles Bell as he was inferior to him in a minute acquaintance with general anatomy. And on one point we are sure that all who are conversant with the works of Galen will bear us out in our assertion, that no man could have reasoned more correctly from the facts ascertained in his time, or have drawn conclusions more legitimately than he does from the premises then set down as established. We wish indeed we could say that the masters of our profession in the present time were as well initiated in the science of abstract reasoning, and were as familiar with the principles of the higher philosophy, as they were in the days of Galen, seventeen hundred years ago. But the truth is that with many advantages over antiquity the present age seems to labour under this disadvantage, that scientific truth not being based as in former times on philosophical arrangement, it but too often happens that all our knowledge is merely an aggregate of particular facts or ideas, and that the mind, by increasing its store, does but add to its own perplexity.

The Second Book of Theophilus, which is an abridgment of the fourth and fifth of Galen's work, contains a description of the structure and uses of all the abdominal viscera. In this region are seated the four natural or nutritive faculties with which all living organized bodies are endowed, the Attractive, the Retentive, the Alterative, and the Expulsive. For a full exposition of the ancient opinions on this head, we would refer to Galen's work "On the Natural Faculties;" Marcrobius, *Saturn.* vii. 4; Haly Abbas, *Theor.* iv. Avicenna I. F. I., *doc.* 6, and Actuarius, *Meth. Med.* vi. 7. Instead of indulging in long quotations from these authors, we shall give an illustration of the ancient doctrine from the works of a learned modern writer, Fernel, who appears to have been thoroughly acquainted with the principles of the ancient physiology:

"If we consider any kind of food, such as bread, flesh, fruit, or wine, it will be seen that it differs much from the nature of our bodies, nor could be converted into its substance or assimilated to it but by undergoing great changes. Nor can it be all altered by digestion, for however good and suitable the food may be, it contains one part fit for nourishing the body, and another part unfit for this purpose. This latter portion must be *expelled* from the body lest it should prove deleterious, wherefore there must be in us some power or faculty for removing this nuisance. The useful part of the aliment which is left, and is fitted for nourishing the body, unless it be brought to all parts of the body cannot nourish them, but as it cannot go to them of its own accord, it must be drawn and *attracted.* When attracted and joined to it, unless it is retained for some time it cannot be concocted, for whatever is dragged hither and thither, does not easily undergo concoction. Wherefore it is necessary that there should be in us some power to *retain* the element." (*De anim. Facult.* c. 4.)

The Arabians, as for example Averrhoes, and their copyist Actuarius, added to the faculties already enumerated, the Discriminative, by which they appear to have meant (to use the language of Dr. Paris,) "*the selecting tact* with which the lacteals are endowed, and which enables them for a considerable time to reject imperfectly formed or vitiated chyle." (On Diet., p. 220.) Theophilus is at great pains to point out the existence of these faculties in the case of the stomach and of the uterus, between which he institutes an ingenious comparison, (c. 5, 6.) If the reader would wish to see the objections which may be stated to the ancient doctrines with regard to the natural faculties he may consult Dumas, (*Principes de Physiol.* t. i, p. 21,) who, like many of his contemporaries and successors however, would appear to have been but very imperfectly versed in ancient physiology.

The ancient physiologists held that the *liver* is the primary organ in the abdominal cavity, all the other viscera situated there being merely subsidiary to it. In short, all the Greek and Arabian physiologists, accounted the liver to be, as Galen expressed it, the primary organ of sanguification, the viscus by which the food is converted into *blood*, that universal pabulum from which all the other parts of the body are constructed. Thus, the stomach was held to be the viscus in which the food is melted down and reduced to a state adapted for absorption, in which condition it is attracted by the liver, and there mainly is converted into blood, to which office the spleen also contributes by absorbing the more recrementitious part for its nourishment; part likewise passing off by the gall-bladder, and another portion by the kidneys. As Nemesius explains it, the spleen draws off the lees of the blood, the gall-bladder the bitter part, and the kidneys the serum. (§ 23.)

Hippocrates, Aristotle, Galen, and in a word all the authorities agree that the *stomach* performs the same office to an animal that the earth does to a vegetable, namely, that it is the store-house from which the organism connected with it draws its nutritious juices. That the stomach is possessed of the faculty of reducing the food to a fluid state they were well aware, but whether the process by which it effects this be analogous to solution or fermentation (πεψις), they were not fully agreed, but in general they held that digestion is a combination of both these processes. "Digestion," says Actuarius, "is performed by moderate heat and moisture." (*De Sp. anim.* ii.) Of the existence of a solvent or *menstruum* in the stomach to which Marcrobius (*Saturnal.* vii. 8,) actually gives the name now applied to it, *ventralis humor* or gastric juice, they were well aware, and although they attributed part of the process to concoction, they recognized it as a vital process, the heat in this case being merely the instrument of the principle of life. "Digestion," says Averrhoes, "is performed by concoction, and concoction by heat, not that the prime mover in the operation is heat, but the nutritive soul." The food being reduced to chyle, all that portion of it which is adapted to the wants of the system was supposed to be absorbed by the *vena portæ*, called by the ancients *vena truncalis*, from its supposed analogy to the trunk of a tree, the mesenteric and gastric veins being its roots, and the hepatic its branches. In these the chyle puts on the appearance of the substance of the liver, that is to say, is converted into blood, which is distributed over the system by the veins and arteries.

The following description of the process by an ancient author, but the last in the list of the ancient physicians, we mean Actuarius, may be interesting to some of our readers :

"The food in the stomach being altered and concocted, when the meseraic veins, which derive their origin from the liver, have, by their vein called ramalis, sucked the stomach and intestines, and have emulged, as it were, the purer part, (namely the food converted into chyle,) drawing it, as it were, through a strainer, they convey it to the concave part of the liver, and deliver it over to its sanguificatory power. Here then if nothing impede it, when it is changed into blood, whatever is subtile and acrid is received by the gall-bladder, which is placed at the convex part of the liver, and attracts the bile; but whatever the blood possesses of a terrene and melancholic humour, is attracted to the spleen by some natural faculty, whereby every part attracts whatever suits its nature. Thirdly, however, the serous humour remains. It is attracted by the kidneys." (*De Urinis.* § 4.)

From what has been stated it will be seen that the ancient physiologists held the spleen to be a portion of the liver, or at least, that both viscera combine together to the performance of one common function. Aristotle's reasoning on this head has always appeared to us singularly ingenious, we would almost say, completely conclusive :

"Of the viscera some appear to be single or solitary, as the heart and lungs; some double, as the kidneys; and some of a doubtful nature. The liver and spleen would seem to be of the doubtful nature, for each of them would seem to be a single viscus, and yet both to be possessed of a similar nature for one common purpose. In fact all the viscera are double, the cause of which is the division of the body into two parts united together for one common end. For it is divided into upper and lower parts; into anterior and posterior; and into right and left. Therefore the brain of all animals is double, and so is each of the senses. By the same analogy the heart has its cavities; and in oviparous animals the division of the lungs is so great, that they might seem to be two lungs. The case of the kidneys is quite obvious. But regarding the nature of the liver and spleen, doubts may be entertained. The reason of this would seem to be that in those animals which decidedly have a spleen, it would appear to be a spurious liver; but in those which have it not decidedly but only a very small one, by way of imitation, as it were, the liver evidently appears to be composed of two parts, the larger portion of which is situated in the right, and the smaller in the left side. And in oviparous animals, although less apparently than in viviparous, the liver is clearly divided: as in certain countries hares have two livers, and in like manner certain fishes, such as the cartilaginous. But because the natural position of the liver is on the right side, the nature of the spleen hence becomes apparent; for it is necessary, but not indispensably necessary..... The liver and spleen assist in the concoction of the food." (*De Partibus animalium*, iii., 7.)

Almost all the Greek physiologists, Galen and Theophilus included, adopt the views of Aristotle. Galen calls the spleen an imitation or resemblance of the liver; and Averrhoes, the Arabian, holds the spleen to be a second liver. (*Colliget* i. 9.) We may remark here that although modern physiologists are greatly divided in opinion regarding the functional office of the spleen, many of them concur with the ancients in holding that the spleen is a portion of the hepatic system. For instance, Blumenbach says, "since the spleen is the only organ of that description quite destitute of an excretory duct, excepting its veins which run ultimately to the liver, its function is probably subservient to the latter. This opinion has appeared strengthened by the observations that in animals deprived of their spleen, an experiment made from the most remote period, the cystic bile is sometimes found pale and inert." (Physiology, § 26.)

Little need be said respecting the views entertained by the ancients with regard to the other abdominal viscera, seeing they differed but little from those of the moderns. They were aware that the reason why the intestinal canal is so much prolonged and convoluted, is in order that the chyle might lodge in it and be presented to the mouths of the mesaraic vessels, and by them be conveyed to the liver. Erasistratus, Herophilus, and Galen were acquainted with the lacteals. (See Galen *De Admin. anat.*, *num sang. in art. cont.*) Galen describes the *pancreas* (*de ven. et art. dissect.* c. i. and *de U. P.* v. 2;) but Theophilus would seem to be guilty of the mistake of confounding it with the mesentery, pp. 77, 15. (See Dr. Greenhill's note.) The epiploon, says Aristotle, is of a fatty nature, and being warm contributes to digestion. (*De Partibus anim*, iv. 3.)

The kidneys, it was held, possess the faculty of attracting the watery portion of the blood to them. (Aristotle *de part. anim.* iii. 7-8, Avicenna, lib. iii. Fen. 8. t. i.) This they were supposed to do direct, either by their veins or by certain undiscovered pores, the existence of which is not admitted by modern anatomists.

Theophilus concludes the chapter on the organs of nutrition with an elaborate recapitulation of the processes which the food undergoes from its entrance into the mouth, to its complete assimilation to the substance of the body. Simplicius states them more briefly, thus: "it is the teeth which masticate the food, the stomach which digests it, and the liver which converts it into blood." (*Comment. in Aristot. Auscult. Nat.* ii.)

The THIRD BOOK of Theophilus treats of the parts seated within and upon the thorax. It is abridged principally from the sixth and seventh books of Galen's work. Galen and the other physiologists after him, held that the cavity of the thorax is intended solely for the reception of the *heart*, all the other organs situated there being merely assistants which minister to its purposes. The heart, in a word, was accounted the seat of the vital powers, the fountain of life, and the stove from which the innate heat is diffused over the system by the arteries. "The heart," says Aretæus, "is the origin of life and respiration, for it imparts to the lungs the desire of drawing in cool air." (*Morb. Acut.* ii. s.) Averrhoes, the Arabian, compares the heart to a furnace, the animal heat to the flames, and the vital principle to the person who supplies it with fuel. (*In Cant. Avicen.* Tr. ii.) He says in another place, that the heart is the primary organ of the body, and the *ultimum moriens.* (*Collect.* i. 21.) The heart therefore is the distinguishing organ of the higher classes of animals, in the more perfect of them maintaining a high degree of temperature, whereas in the lower the innate heat is languid and inert like that of plants, and equally diffused over all the body. (Aristotle *de Part. Animal.* iv. 6.) The earlier physiologists and philosophers differed much in opinion regarding the uses of respiration to the animal frame. Asclepiades, according to Galen, (*de Usu Respirat.*) held that it is for the generation of the soul itself, breath and life being thus held to be identical; for the strengthening of it according to Praxagoras; for the refrigeration of the innate or animal heat, according to Philiston, Diocles, and Aristotle; for its nutrition and refrigeration, according to Hippocrates; for the filling up of the arteries with spirit, according to Erasistratus. All these opinions are discussed and commented upon by Galen, who comes to the conclusion that the purposes of respiration are two-fold; first, to preserve the animal heat, and second, to evacuate the fuliginous part of the blood. (Ibid.) He points out the analogy between respiration and combustion, and comes to the conclusion that the processes are of a similar nature; he therefore compares the lungs to a lamp, the blood to the oil, the heart to the wick, and the animal heat to the flame. (Ibid. 3.)* He speculates acutely regarding the cause why air in certain vaults which have been recently plastered with lime, and in apartments containing the fumes of extinguished charcoal, proves suddenly destructive to life, and determines the cause to be that the air in these cases acts upon the lungs in the same manner that the torpedo acts, namely, by superinducing a certain torpor or prostration of the powers.

Galen, and his epitomist, Theophilus, have given, upon the whole, a wonderfully accurate description of the substance, the cavities, and the valves of the heart, and also of the arterial and venous systems. With regard to the substance of the heart, Galen hesitates to admit with Hippocrates that it is muscular, stating, as is really the case, that its fibres differ considerably from those of muscles. (*De Motu Muscul.* § 3.) Galen everywhere inculcates that the heart is the centre of the arterial system, and that the left side attracts spirits from the lungs and blood from the right ventricle, and sends them mixed together to all parts of the system. The office of the valves of the heart is to prevent the regurgitation of the blood from the left to the right side of the thorax during the systole. On all occasions he maintains against Erasistratus that the contents of the arteries is blood and not spirits, and as he further was acquainted with the anastomosis of arteries and veins, it must be admitted, (and, in fact, it is admitted by Harvey himself,) that Galen had made a very near approach to the Harveian theory of the circulation. We are inclined to believe that he held (although we must admit that in no part of his works has he given so clear an exposition of his system as could have been wished,†) that at every systole of the heart and arteries a certain portion

* The readers will at once perceive that this is an anticipation of the theory of respiration lately announced by Liebig.

† The passages in which Galen expounds his views with regard to the functional offices of the heart are so widely scattered over his works that it would cost much time and trouble for us to prove by quotations the correctness of the exposition which we have given of his system. Our readers therefore must be content to take our word for it that our opinions are formed from a careful study of his works. The following passage from the works of Averrhoes will show that the views entertained by the Arabians were scarcely of their contents is thrown out by the exhalent and secretory vessels, and that at every diastole blood from the liver and air from the lungs rush in to supply the *vacuum.* It thus appears that, like certain physiologists of the present day, he held that the expansion of the ventricle and arteries is the cause of the influx of the blood, instead of maintaining, like Harvey, that the influx of the blood causes the diastole. But the grand point of difference between Galen and Harvey, and upon which the celebrated theory of the latter mainly rests, is the question whether or not at every systole of the left ventricle more blood is thrown out than what is expended on exhalation, secretion, and the nutrition of the body. Upon this point Galen held the negative, Harvey the affirmative.*

On no point of the circulation has there been greater diversity of opinion than with regard to the power which brings the blood to the heart from all parts of the body. Some with Harvey hold that the blood is propelled by a *vis a tergo;* others contend for a suction power in the right ventricle; whilst others explain the movement upon the principle of atmospheric pressure, and the tension of the fasciæ, muscles, &c. In the remarks of Galen, contained in the following passage, will be found the germs of most modern theories on this head:

"The heart itself having every imaginable power of attraction, receives into its cavities the materials which flow to it, seizing upon and, as it were, *sucking* them in. Whether it be that as the bellows of smiths when expanded suck in the air, so does the heart much more: or whether as the flames of the lamp attract the oil, so neither is the heart destitute of such a power, being the source of innate heat; or whether as the magnet by some alliance of properties attracts the iron: for what is more nearly allied to it than air for cooling it? or what more necessary than blood for nourishing it?" (*De U. P.* vi. 15; *de Nat. fac.* iii.)

Avicenna expresses himself in similar terms: "the attraction is performed either by some attractive power as the magnet attracts iron; or by filling a *vacuum* as water is drawn into a syringe; or by heat as the at all different from those we have ascribed to Galen: "Nutriment comes to the heart by the vein which connects it with the liver, the valve which is in that auricle being so constructed that when opened it may furnish an entrance to the blood, and when shut that it may retain it. But the other ear, [right ventricle?] which, as some suppose, is from the vein that carries blood to the lungs for the sake of nutriment, [for no veins are continuous with the lungs,] has its valve so constructed thut it may furnish outwardly an open passage to the lungs; but the other of these cavities, [left ventricle?] is furnished with a three-fold valve, [mitral?] so that when opened blood and spirits may flow inwardly into the arteries, nor can it regurgitate. This is in the left ventricle of the heart, as is the orifice of the great artery. But the other ear, [left auricle,] which is in the other part, is the mouth of the artery, [pulmonary veins?] which goes to the lungs, by which the ventilation of the blood is performed; wherefore its valves are so formed as to open outwardly." (*Collectan.* § i. c. 9.)

* In proof of the fact that a considerable contraction of the cavity of the ventricle takes place in its systole, Harvey appeals to observations made on the hearts of snakes dissected alive. (*De motu Cordis*, c. x.) The writer of this paper has repeatedly dissected vipers in order to satisfy himself in regard to the fact assumed by Harvey; but he must say that even with the assistance of a microscope, neither himself nor certain friends whom he invited to assist him in forming a judgment in the matter, could ever perceive any such contraction of the bulk of the heart in systole as would have led them to suppose that any sensible portion of its contents had been thrown out. The pulsations of the heart were marked indeed by some change of colour and of tension; but we repeat that we never could detect any appearance of a sensible contraction of the ventricle, or of a current of blood passing along the arterial tube.

oil in the lamp is attracted, although this case may also be referred to the filling of a *vacuum*." (Lib. i. *Fen*. i. Doc. vi. c. 3.)

The Third Book of Theophilus contains upon the whole a pretty correct abstract of Galen's doctrines which we have been discussing, and also treats briefly of the organs of speech, and of lactation. But we must pass on to the Fourth Book, in which is contained the substance of the eighth, ninth, tenth, and eleventh Books of Galen, relating to the Head. Theophilus opens the subject thus :

"That there are three primary organs which regulate the animal frame, namely, the brain, the heart, and the liver, is admitted by all wise physicians; and that the brain by the nerves conveys sensation and motion to all the body, and the heart ventilating the whole body, by the arteries, thereby animates it, and that the liver by the veins, as by certain canals irrigates the members of the body and thereby nourishes and enlarges them, is admitted not only by the skilled physician, but also by the vulgar."

The primary office of the brain, then, was held to be, to impart motion and sensibility to all parts of the body by the nerves. It would appear from the Manual of Anatomy, by Rufus Ephesius, that the celebrated Erasistratus had attempted to classify the nerves into the nerves of sensation and of motion; but Galen undoubtedly has the merit of having first established this theory, so that it was admitted by all the subsequent authorities on medicine both Greek and Arabian, nay even by Galen's great modern antagonist Vesalius. Galen divided the brain into two parts, an anterior called the cerebrum, and a posterior called the cerebellum; the former of which, he says, is of a soft nature and gives rise to the sensatory nerves, and the latter is hard and gives rise to the motory. Without wishing in the slightest degree to detract from the merit of the late Sir Charles Bell, and the other physiologists, English, French, and Italian, who have improved this branch of physiological anatomy, we must be permitted to say that, although there be a portion of error mixed up with truth in Galen's theory, it enabled him to classify the nerves of the face, the neck, the tongue, and of the organs of nutrition and respiration, with a degree of accuracy and discrimination such as will hardly be credited by those who think very meanly of the Galenical anatomy, and would restrict all knowledge of the subject to the present age.

Galen's description of the brain is much the same as that usually adopted by modern anatomists, until Reil, Malacarne, Tiedemann, and other labourers in the same department, extended our knowledge of its minute anatomy. Theophilus describes the cerebral vapour contained in the ventricles as being thin, and free from impurities; but does not seem to have been aware that it is a fluid in its natural state. He thus describes the office of the animal spirits:

"Deservedly the brain has received the uppermost region of the body for its abode, like some master of a house, having as its guardsmen all the senses, the sight, the hearing, the smell, the taste, and the touch; which despatching over the whole body, she confers sensibility upon it, imparting to it sensation and motion by the animal spirits."

Before concluding this part of our subject we would wish to say a few words respecting the opinions entertained by the ancients as to the connexion between the mind, and certain organs of the human frame. Plato divided the soul of man into three distinct parts, reason, passions, and desires, (see in particular *De Repub.* lib. iii.) Viewed as the division of a material object this arrangement must appear highly fanciful, not to say absurd; but as it is illustrated by the learned Fernel from the formation of the posterior geometrical figures from the first it appears in a different light. Thus, if a triangle be properly applied to another triangle it will form a tetragon, which is not a composite but simple figure; and if another triangle be added to it a pentagon is formed, which is simple in reality but composite in power. (*De Anim. Facult.* c. 18.) Galen, whose mind was deeply imbued with the principles of the Platonic philosophy, applies this doctrine to the human body, maintaining that the brain is the seat of reason, the heart of the passions, and the liver of the desires. (*De Facult. naturalibus, passim.*) There is scarcely a speculative opinion in science or philosophy which ever obtained a more general and enduring acceptance than this doctrine obtained, having been adopted generally by all the Greek *sçavans* after Galen, by the Arabians, and by the earlier modern authorities, as for example, Fernel (*de Anim. Facult.* c. 14.) Nay, very recently the celebrated Bichat espoused this doctrine, though without acknowledging the quarter from whence he derived it. He argues that general observation has proved that during intense passions it is the *heart* that is primarily affected, producing palpitations, &c., that the actor in representing such emotions applies his hand to his heart and not to his head. (*Sur la Vie et la Mort*, p. ii. a. 5, and further p. 227.) In like manner Dr. Mason Good argues that the vulgar character of the *liver* as indicative of hatred and revenge is not merely figurative, but has a foundation in nature; that anger long indulged is known to affect the functions of the liver, and has often laid the foundation for jaundice; and that the seat of anger has in the poetical language of most countries been transferred to this organ, and that choleric and irascible are convertible terms in the popular language of our day. (Study of Med. vol. iii. p. 114.)

There occurs in Theophilus (p. 184.) another division of the brain as regards its connexion with the operations of mind to which we would wish to direct attention, as bearing a considerable resemblance to that which is adopted by the more scientific phrenologists of our times. According to it that department of the mind called the fantasy, namely the sensorium or receptacle of ideas, is connected with the anterior part of the brain; cogitation or the *discursus mentis* with the middle; and memory with the posterior. We shall attempt a translation of this passage in the work of Theophilus:

"The anterior ventricles of the brain comprehended under the forehead, contain the fantastical part; for there being three general functions performed by the vital spirit, namely, fantasy, memory, and the *discursus mentis*, three distinct places of the brain are allotted to them for their abode, the anterior, the posterior, and the middle. The fantasy is seated in the anterior part, the *discursis mentis* in the middle, and the memory in the posterior. But how and in what manner Homer says that the ruling part of the soul is in the heart, and also many other Greeks, nay even the divine gospel itself, 'for why,' says it, 'do thoughts arise in your hearts?' (Luke, xxiv. 38,) I cannot tell, for often physicians inquiring concerning the loss of reason and memory, in order to find out the part affected, could discover none except the brain. Wherefore all suitable applications for the cure, and fomentations, and other remedies, they apply to the head and not to the heart," &c.

Now it is worthy of remark, that no traces of this hypothesis are to be found in the works of Galen. It originated, no doubt, in the eastern schools of philosophy, and accordingly the first indication of it, as far as we know, is to be found in the works of Nemesius, who is said to have been Bishop of Emesa in the fifth century. It is adopted by all the Arabian medical authors. (See in particular Averrhoes, *Colliget.* ii. 20.) We need scarcely stop to point out the resemblance between this division of the brain, and that of the modern phrenologists, who hold that the intellect is connected with the anterior part, the moral feelings with the middle, and the animal appetites with the posterior.

We have already taken up so much space with the analysis of the work before us, that we must be very brief in noticing the other subjects handled by Theophilus. He treats of the nose, the eye, its appendages, the tongue, the jaws, the teeth, and the ears. We would beg to direct the attention of our readers more particularly to Galen's admirable disquisition on the eye. (*De U. P.* x.) It is singular that he should have thought the crystalline lens the seat of vision, notwithstanding that he appears to have been familiar with the operation of couching the cataract. (*Ars Medica*, and *de U. P.* x. 1.) The ancients in general looked upon the cataract as being a film anterior to the lens; some of them, however, were aware that the disease is sometimes seated in the lens itself. (See notes to the English translation of Paulus Ægineta, by Mr. Adams, p. 393.)

The contents of the Fifth and last Book of Theophilus are mostly taken from the twelfth, thirteenth, fourteenth, fifteenth, and sixteenth books of Galen's work, and from Hippocrates *De geniturâ* and *De naturâ pueri.* He treats first of the spine, beginning with the spinal marrow, which, according to Galen, springs from the brain as a trunk, and is divided into nerves, as it were into branches. He then touches briefly on the vertebræ and the construction of the various joints of the body. From the seventeenth book to the end he dwells upon the subject of generation, anatomically and physiologically, and handles it in the fullest manner. Though Galen and Theophilus are sometimes guilty of mistakes in their anatomical descriptions, from applying to the human body what is applicable only to the inferior animals, the general idea which they give of the relation between the sexes is correct, and corresponds with the comparison made by many modern authors, namely, that all the parts in the two sexes are analogous, with this difference, that in the male they are placed without, whereas in the female they are situated within. Most of the ancient anatomists fall into the mistake of describing the human uterus as consisting of two distinct cavities, a right and a left. But, as Dr. Kidd points out, there is a passage in the works of Galen from which we must infer that he had more correct ideas on this head.* The same cannot be said of Theophilus, who, in his Commentary on Hippocrates, dis-

* Vol. v. p. 789, ed. Kuhn. See Kidd's Analysis of Galen's works in the Transactions of Provincial Association.

tinctly says that the womb consists of two cavities, "separated by a certain membrane." (See Greenhill's *Adnotationes*, p. 330.)*

At c. 33, Theophilus gives a brief description of the development of the fœtus *in utero;* it is, however, but a meager abstract of Galen's ingenious tract "on the formation of the fœtus." Galen there states that it had been made a question what part of the body is first formed, some contending for the priority of the heart and others for the liver. Many of the Stoic and Peripatetic philosophers were of the former opinion, principally, as he says, on theoretical grounds. Galen maintains, from observation and dissection, that the liver has an existence antecedent to the heart, and yet he does not hold that even it is the part of the fœtus which is first organized. First of all, he says, are formed the vessels that connect the fœtus with the uterus, being analogous to the roots of trees; these unite together and form the umbilical vein, which is analogous to the trunk, and its ramifications to the branches. He would appear to refer the first organization of the fœtus to what Blumenbach calls a "nisus formativus," which he calls a power in the semen. He argues ingeniously that it is not correct that the parts first formed produce those which are next in the order of formation; but that nature first forms one part of the fœtus and then another, and does not as it were set the work a going and then leave the parts just made to complete the work. Upon the whole then he does not approve of the views of those physiologists who hold that the liver forms the heart and the heart the brain, but maintains that these three primary organs have a mutual dependence upon one another, and that in like manner the secondary organs depend upon the primary. He concludes with confessing the difficulty he felt in determining what the substance of the soul is which presides over the formation and preservation of our bodies, in which as all recognize the traces of intense skill, some referred them immediately to the work of an intelligent principle, whilst others hold that the principle which directs the process is itself devoid of understanding.

But it is now time for us to draw to a conclusion. We trust that the exposition we have given of the doctrines entertained in former times by our profession on so many important subjects, will prove not altogether devoid of interest, even to such of our readers as may think that we have paid greater deference to the opinions of the ancients than they actually merit. It surely can neither be uninteresting nor unprofitable to compare the opinions entertained in the present advanced state of physiological science, with those which occurred to the minds of the great fathers of medicine. A perusal of old doctrines, to use the words of a learned author, may afford amusement, if it be true "that what from its antiquity is but little known, has from that very circumstance the recommendation of novelty." (Harris, *Philosophical Arrangements*, p. 111.)

* Ib. On the other hand the description of the uterus given by Moschion is very correct.

